# OpaR Exerts a Dynamic Control over c-di-GMP Homeostasis and *cpsA* Expression in *Vibrio parahaemolyticus* through Its Regulation of ScrC and the Trigger Phosphodiesterase TpdA

**DOI:** 10.1128/spectrum.00872-23

**Published:** 2023-05-18

**Authors:** David Zamorano-Sánchez, Jesús E. Alejandre-Sixtos, Adilene Arredondo-Hernández, Raquel Martínez-Méndez

**Affiliations:** a Programa de Microbiología Genómica, Centro de Ciencias Genómicas, Universidad Nacional Autónoma de México, Cuernavaca, Morelos, Mexico; Navarrabiomed-Universidad Pública de Navarra (UPNA)-Complejo Hospitalario de Navarra (CHN), IdiSNA

**Keywords:** *Vibrio parahaemolyticus*, biofilms, c-di-GMP, gene-regulation, signal transduction

## Abstract

The second messenger cyclic dimeric GMP (c-di-GMP) plays a central role in controlling decision-making processes that are vitally important for the environmental survival of the human pathogen Vibrio parahaemolyticus. The mechanisms by which c-di-GMP levels and biofilm formation are dynamically controlled in V. parahaemolyticus are poorly understood. Here, we report the involvement of OpaR in controlling c-di-GMP metabolism and its effects on the expression of the trigger phosphodiesterase (PDE) TpdA and the biofilm-matrix related gene *cpsA*. Our results revealed that OpaR negatively modulates the expression of *tpdA* by maintaining a baseline level of c-di-GMP. The OpaR-regulated PDEs ScrC, ScrG, and VP0117 enable the upregulation of *tpdA*, to different degrees, in the absence of OpaR. We also found that TpdA plays the dominant role in c-di-GMP degradation under planktonic conditions compared to the other OpaR-regulated PDEs. In cells growing on solid medium, we observed that the role of the dominant c-di-GMP degrader alternates between ScrC and TpdA. We also report contrasting effects of the absence of OpaR on *cpsA* expression in cells growing on solid media compared to cells forming biofilms over glass. These results suggest that OpaR can act as a double-edged sword to control *cpsA* expression and perhaps biofilm development in response to poorly understood environmental factors. Finally, using an *in-silico* analysis, we indicate outlets of the OpaR regulatory module that can impact decision making during the motile-to-sessile transition in V. parahaemolyticus.

**IMPORTANCE** The second messenger c-di-GMP is extensively used by bacterial cells to control crucial social adaptations such as biofilm formation. Here, we explore the role of the quorum-sensing regulator OpaR, from the human pathogen V. parahaemolyticus, on the dynamic control of c-di-GMP signaling and biofilm-matrix production. We found that OpaR is crucial to c-di-GMP homeostasis in cells growing on Lysogeny Broth agar and that the OpaR-regulated PDEs TpdA and ScrC alternate in the dominant role over time. Furthermore, OpaR plays contrasting roles in controlling the expression of the biofilm-related gene *cpsA* on different surfaces and growth conditions. This dual role has not been reported for orthologues of OpaR, such as HapR from Vibrio cholerae. It is important to investigate the origins and consequences of the differences in c-di-GMP signaling between closely and distantly related pathogens to better understand pathogenic bacterial behavior and its evolution.

## INTRODUCTION

The genus *Vibrio* includes several bacterial aquatic species that continuously threaten human health worldwide. Vibrio parahaemolyticus is one of the most common etiological agents of gastroenteritis caused by raw seafood consumption (1, 2). This pathogen also impacts human activities such as shrimp farming due to its ability to cause acute hepatopancreatic necrosis in these organisms (3, 4). The ability of this pathogen to adapt to the ever-changing ecosystems in which it dwells is of paramount importance for its colonization success and its persistence and dissemination in the environment.

As in many pathogens, it is likely that the ability of V. parahaemolyticus to form complex multicellular associations within a self-produced exopolymeric matrix, known as biofilms, plays a key role in its ability to resist adverse conditions such as antimicrobial insults and nutrient scarcity (5, 6). Our knowledge of the biofilm formation process and its regulation in the *Vibrio* genus has been mostly obtained from study of the human pathogen Vibrio cholerae (7). Although several of the paradigms of biofilm formation in V. cholerae are true for other *Vibrio* species, important particularities in biofilms within the *Vibrio* genus justify the need to study them on a case-by-case basis (8). Increasing our understanding of the process of biofilm formation could enable more educated design of contention strategies to combat V. parahaemolyticus outbreaks.

One general element of study within the field of biofilm formation and regulation is the role of the second messenger cyclic dimeric GMP (c-di-GMP). This intracellular messenger plays a central role in the decision-making process in bacteria, including the choice between a planktonic or a sessile lifestyle (9, 10). c-di-GMP is synthesized by diguanylate cyclases (DGCs), which have a catalytic GGDEF domain, and it is degraded by specific phosphodiesterases (PDEs) with a catalytic EAL or HD-GYP domain (9). c-di-GMP signaling modules are typically composed of DGCs, PDEs, and one or multiple effectors that can interact with c-di-GMP and consequently alter a physiological response (11). Changes in c-di-GMP levels affect the transcriptomic profile of V. cholerae and its ability to form biofilms (12, 13). Multiple PDEs and DGCs have been shown to participate in controlling the intracellular pool of c-di-GMP in V. cholerae; however, less is known about the cues that result in changes to the activity or abundance of the components of these c-di-GMP signaling modules (12, 14–16). One key modulator of c-di-GMP accumulation and biofilm formation in V. cholerae is the quorum-sensing master regulator HapR, which negatively controls these processes through its ability to affect the transcription of genes related to c-di-GMP metabolism and exopolysaccharide biosynthesis (14, 17–19). This regulator is conserved in species within the *Vibrio* genus; in V. parahaemolyticus, this regulator was named OpaR due to its involvement in controlling colony opacity, a phenotype influenced by the ability to produce a capsular polysaccharide, CPS. CPS biosynthesis depends on the *cpsA*-*K* genetic cluster, which is regulated positively by c-di-GMP through a hierarchical cascade of transcriptional regulators that are also c-di-GMP receptors (20–22). CpsS negatively regulates the expression of CpsR, a positive regulator of CpsQ which, in conjunction with paralogous proteins such as ScrO, controls CPS production (21, 22). In contrast to HapR, which has been shown to repress exopolysaccharide biosynthesis and c-di-GMP accumulation (17, 23), OpaR was shown to be required for CPS production and c-di-GMP accumulation in the V. parahaemolyticus strain BB22 (21, 24, 25). The absence of *opaR* in strain BB22 does not eliminate biofilm formation but rather affects the biofilm architecture and potentially delays biofilm dispersal or detachment in the liquid-solid interface (24). Recent reports have indicated that OpaR can repress biofilm formation and c-di-GMP accumulation in the V. parahaemolyticus strain RIMD2210663 (26). However, the involvement of OpaR in biofilm formation in strain RIMD2210633 was described only at one time point, while for strain BB22, the kinetics of biofilm formation were evaluated. Furthermore, the biofilm growth conditions were different in both reports. For these reasons, it is unclear whether the apparent discrepancies between these two strains are due to strain variability or a bipolar role of OpaR in regulating biofilm formation, *cpsA* expression, and c-di-GMP accumulation in response to environmental cues.

In a previous report, we characterized a novel trigger phosphodiesterase in V. parahaemolyticus which we named TpdA (27). Trigger phosphodiesterases are c-di-GMP effectors which have enzymatic and regulatory activities that are typically anticorrelated (28). The enzymatic activity of TpdA anticorrelates with its ability to activate its own transcription (27). The ability of TpdA to promote its own transcription when c-di-GMP levels drop could accelerate c-di-GMP depletion or extend its duration. On the other hand, preventing TpdA expression might be necessary to set a c-di-GMP baseline that favors the rapid onset of biofilm formation in response to favorable conditions. The absence of TpdA has an effect on intracellular c-di-GMP levels, but there have been no reports on how this compares to the influence of other important PDEs such as ScrC and ScrG (29–31). These comparisons are key to understanding how c-di-GMP can be controlled in a dynamic fashion.

Our goal in this work was to further characterize the dynamics of c-di-GMP signaling in the V. parahaemolyticus reference strain RIMD2210633. We hypothesized that OpaR, being a key modulator of c-di-GMP homeostasis, would regulate *tpdA* expression (25, 26). Our results uncovered different degrees of influence of OpaR-regulated PDEs (ScrC, ScrG, and VP0117) on c-di-GMP accumulation and *tpdA* expression. We also observed a dominant c-di-GMP-degrading role for TpdA in planktonic cultures compared to ScrC, ScrG, and VP0117. In contrast, ScrC played the central c-di-GMP degrader role for the first 72 h of growth on solid medium until TpdA took over after 96 h. We also provide evidence which suggests that OpaR can both positively and negatively modulate the expression of the c-di-GMP regulated gene *cpsA* under different growth conditions. This yin-and-yang nature of OpaR is likely enabled by its repertoire of regulatory targets, which includes not only PDEs but also DGCs and potentially dual-function DGC-PDEs.

## RESULTS

### The expression level of *tpdA* is altered by the absence of OpaR and OpaR-regulated phosphodiesterases.

**FIG 1:**
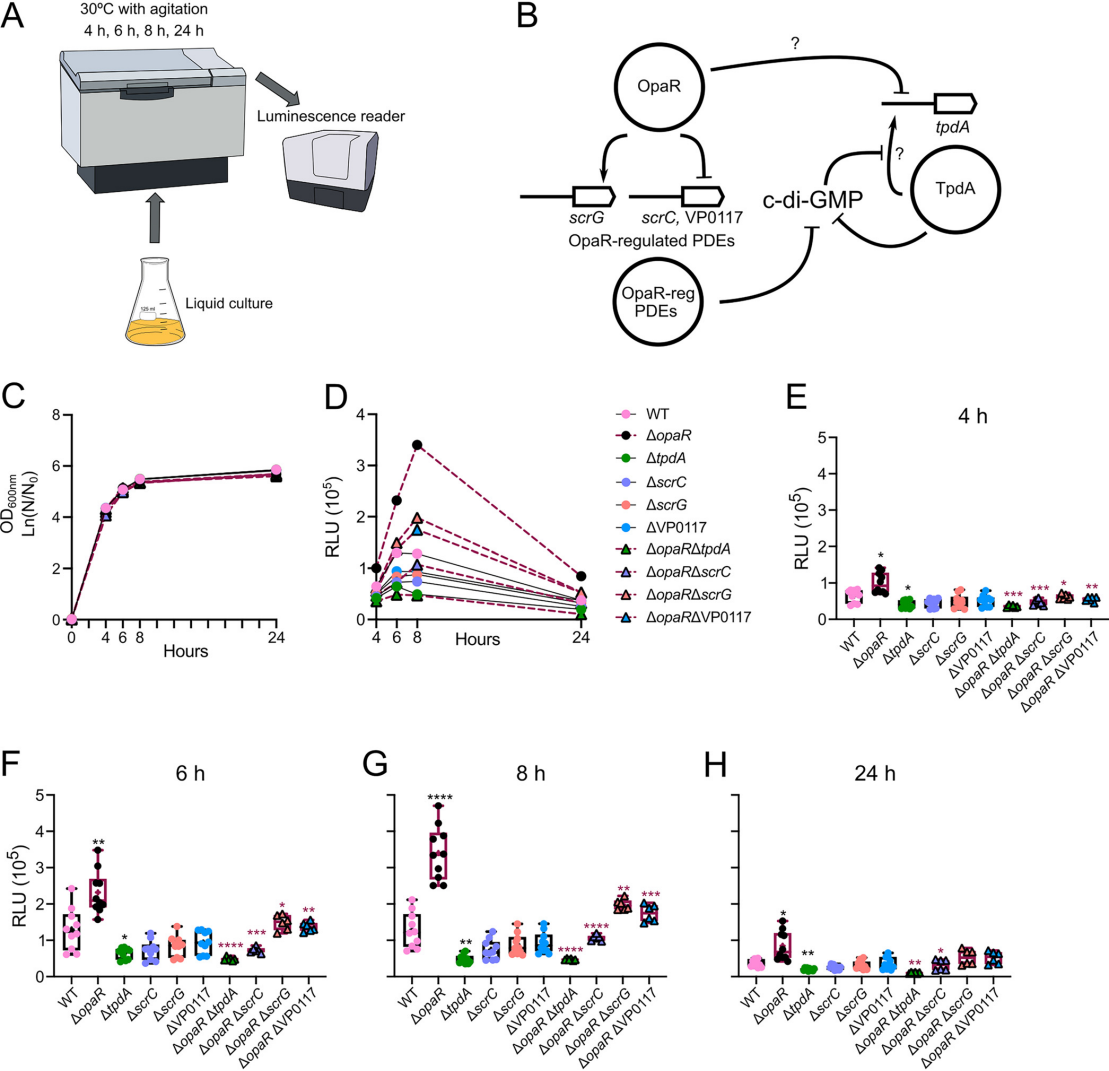
The expression of *tpdA* is oppositely influenced by OpaR and OpaR-regulated phosphodiesterases (PDEs). (A) Schematic representation of the experimental procedure. Cells were grown in liquid cultures with agitation at 30°C. A minimum of six independent biological samples were analyzed, and experiments were performed at least twice. (B) Schematic representation of a proposed model for cyclic dimeric GMP (c-di-GMP) modulation through OpaR and its regulated PDEs, and the potential effect of this modulation on *tpdA* expression. Arrows represent positive transcriptional regulation, while T connectors represent negative transcriptional regulation or enzymatic degradation of c-di-GMP. Question marks (?) indicate that it is unknown whether the regulation is direct or indirect. (C) Mean values for the natural logarithm of the proportion between the current optical density at 600 nm (OD_600_) and the OD_600_ at the start of the experiment. (D) Mean relative light unit (RLU) values of the strains of interest at each time point analyzed. Dispersion of data and statistical analysis are described in panels E to H. Expression values of the wild-type (WT) strain and the single mutants lacking a PDE are connected by a solid black line. Expression values of the strains lacking *opaR* are connected by maroon dashes. (E to H) Box plots representing the expression data, in RLU, of the transcriptional fusion P*_tpdA_*-*luxCDABE* in different genetic backgrounds at 4 time points. Values obtained from WT and single-mutant strains are represented by filled circles of different colors (WT, pink; Δ*opaR*, black; Δ*tpdA*, green; Δ*scrC*, purple; Δ*scrG*, orange; ΔVP0117, blue). Values obtained from the double mutants are represented by triangle symbols which are same color as their corresponding single mutants in the PDE-related genes analyzed. Boxes representing RLU from strains which have the WT allele of *opaR* have black borders; boxes representing RLU from strains in which *opaR* was eliminated have maroon borders. Means were compared using a Brown-Forsythe and Welch analysis of variance (ANOVA) followed by a Dunnett’s T3 multiple-comparison test for direct comparison with the mean of either the WT or Δ*opaR* mutant strain. Black and maroon asterisks (*) indicate statistical differences (adjusted *P* values) compared to WT and Δ*opaR*, respectively. *, *P* ≤ 0.05; **, *P* ≤ 0.01; ***, *P* ≤ 0.001; ****, *P* ≤ 0.0001.

The activity of the P*_tpdA_* promoter is induced by a decrease in c-di-GMP accumulation, in a TpdA-dependent manner (27). Previous reports have shown that the master regulator of the quorum-sensing response, OpaR, regulates several genes whose products are involved in c-di-GMP metabolism (26). To determine whether the presence of OpaR affects the transcriptional activity of the P*_tpdA_* promoter, we mobilized the transcriptional fusion P*_tpdA_*-*luxCDABE*, assembled into the pBBRlux plasmid, to the wild-type (WT) strain and a Δ*opaR* mutant strain. In this assay, light production is proportional to the activity of P*_tpdA_* promoter. We analyzed P*_tpdA_* promoter activity in liquid cultures over time (Fig. 1A) in the WT, Δ*opaR*, and Δ*tpdA* strains, and in strains with deletions in genes regulated by OpaR whose products are PDEs (Fig. 1B) (29–31).

We did not observe significant changes in growth between the strains analyzed (Fig. 1C). Our results revealed a 1.5-, 1.8-, 2.7-, and 2.3-fold change (ratio) in P*_tpdA_* promoter activity in the Δ*opaR* mutant strain compared to the WT strain after 4, 6, 8, and 24 h of growth, respectively (Fig. 1D to H). These results strongly suggest that OpaR negatively regulates the expression of *tpdA*.

As previously shown, the absence of *tpdA* significantly affects the activity of the P*_tpdA_* promoter (Fig. 1D to H). In contrast, the individual absence of *scrC*, *scrG*, or VP0117 did not significantly affect P*_tpdA_* promoter activity (Fig. 1D to H). The absence of *opaR* cannot induce P*_tpdA_* promoter activity when *tpdA* is also absent (Δ*opaR* Δ*tpdA*) (Fig. 1D to H). This strongly suggests that the absence of *tpdA* has a dominant effect over the absence of *opaR* on the activity of the P*_tpdA_* promoter. On the other hand, double-mutant strains which lacked *scrC*, *scrG*, or VP0117 in conjunction with *opaR* showed altered P*_tpdA_* promoter activity compared to the single mutant Δ*opaR* (Fig. 1D to H). The OpaR-regulated PDE which showed the strongest effect on P*_tpdA_* promoter in a Δ*opaR* genetic background was ScrC. The Δ*opaR* Δ*scrC* mutant strain showed lower levels of P*_tpdA_* promoter activity at all time points tested, compared to the Δ*opaR* mutant strain with fold changes of 0.3 to 0.4 (Δ*opaR* Δ*scrC*/Δ*opaR*) (Fig. 1D to H). These results suggest that ScrC is a key contributor to the upregulation of *tpdA* in the Δ*opaR* genetic background, although not the only one. The contribution of ScrG and VP0117 to the elevation of P*_tpdA_* activity in the Δ*opaR* mutant strain was modest (Fig. 1D to H). We observed fold changes of 0.5 and 0.6 when comparing P*_tpdA_* promoter activity in the Δ*opaR* Δ*scrG* and Δ*opaR* ΔVP0117 strains, respectively, to that in the Δ*opaR* mutant strain after 8 h of planktonic growth (Fig. 1G). Together, our results revealed a hierarchical control of P*_tpdA_* promoter activity by OpaR-regulated PDEs (TpdA > ScrC > VP0117 > ScrG).

### OpaR plays a role in maintaining the c-di-GMP baseline during planktonic growth.

**FIG 2:**
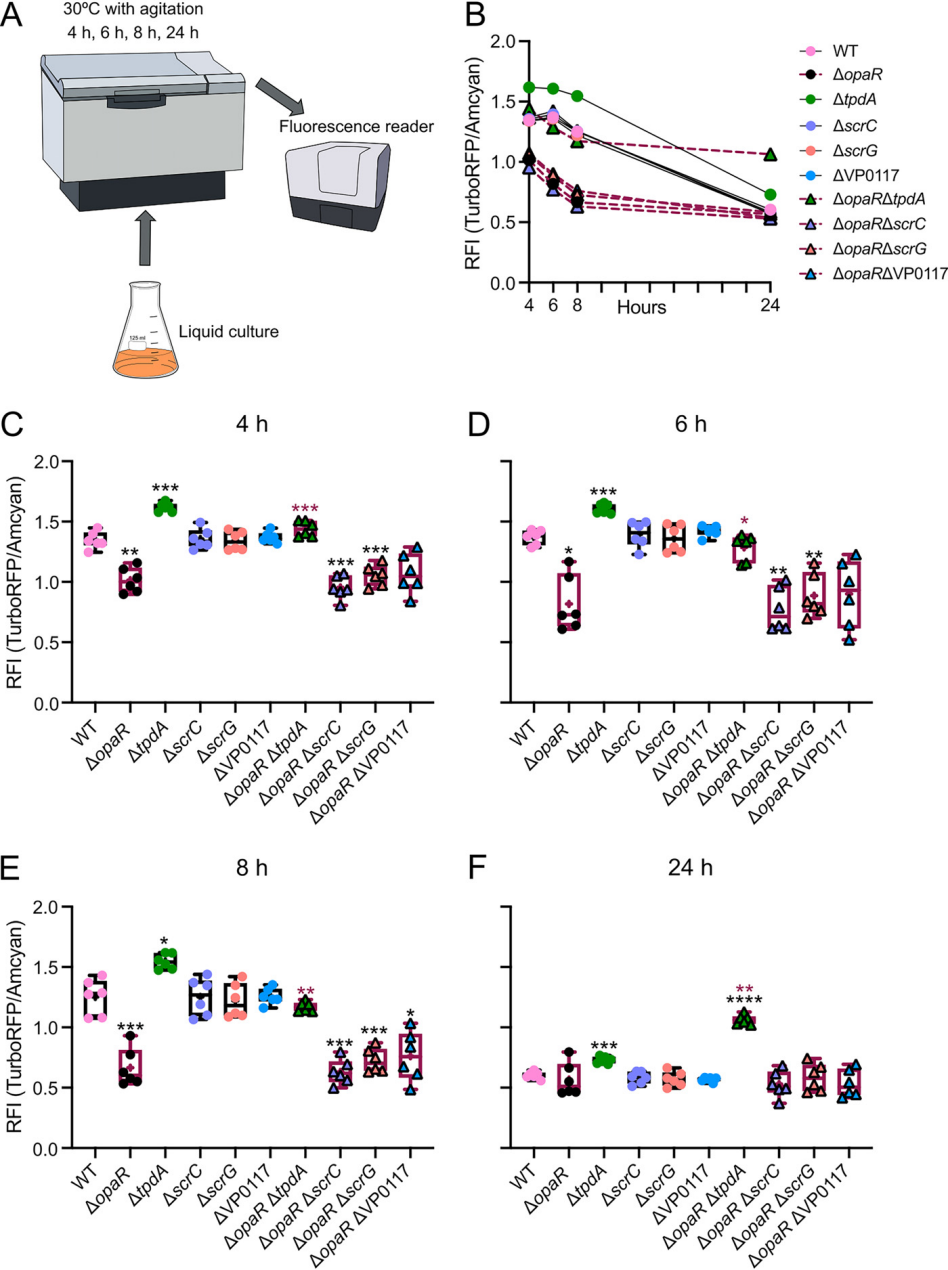
OpaR and TpdA are important modulators of c-di-GMP homeostasis in planktonic cultures. (A) Schematic representation of the experimental procedure. Cultures were grown planktonically with agitation at 30°C. At least six independent biological samples were analyzed across three independent experiments. (B) Mean relative fluorescence intensity (RFI) values of the strains of interest at each time point analyzed. RFI values are proportional to the level of c-di-GMP in the cultures at 4 different time points. Dispersion of data and statistical analysis are described in panels C to F. Symbol color and type and connectors are the same as in the Fig. 1 legend. (C to F) RFI values. Means were compared using a Brown-Forsythe and Welch ANOVA followed by a Dunnett’s T3 multiple-comparison test for direct comparison with the mean of either the WT or Δ*opaR* mutant strain. Black and maroon asterisks (*) indicate statistical differences (adjusted *P* values) compared to WT and Δ*opaR*, respectively. *, *P* ≤ 0.05; **, *P* ≤ 0.01; ***, *P* ≤ 0.001; ****, *P* ≤ 0.0001.

As mentioned previously, the activity of the P*_tpdA_* promoter can be induced by a decrease in c-di-GMP levels (27). Based on this antecedent, we speculated that the induction of P*_tpdA_* promoter activity in the Δ*opaR* genetic background could be due to a decrease in c-di-GMP levels. Furthermore, we also hypothesized that the hierarchical regulation of the P*_tpdA_* promoter by ScrC, VP0117, and ScrG could be explained by differences in c-di-GMP levels in genetic backgrounds which lacked these PDEs. To evaluate c-di-GMP levels in the genetic backgrounds of interest, we used a genetic biosensor previously shown to be able to detect differences in c-di-GMP levels in V. cholerae, V. parahaemolyticus, and Vibrio fischeri (27, 32, 33). In this c-di-GMP biosensor, the production of two fluorescent proteins, AmCyan and TurboRFP, is controlled by a constitutive promoter (32). While AmCyan production is not directly affected by c-di-GMP levels, TurboRFP production is directly controlled by two c-di-GMP riboswitches in tandem (32). Binding of c-di-GMP to the riboswitches favors the production of TurboRFP. Hence, the fluorescence of TurboRFP is proportional to the level of c-di-GMP. AmCyan fluorescence is used to normalize for differences in the abundance of the biosensor or the activity of its promoter. We analyzed c-di-GMP levels in liquid cultures at the same time points, as shown in Fig. 1 (Fig. 2A and B). Our results revealed 0.5-to 0.8-fold changes in c-di-GMP levels when comparing the Δ*opaR* mutant strain and the WT strain during the first 8 h of growth (Fig. 2B to F). After 24 h of growth, c-di-GMP levels in the Δ*opaR* mutant and WT strain were not significantly different (Fig. 2F). This suggests that the absence of OpaR negatively affects c-di-GMP accumulation under these growth conditions.

As previously reported, the Δ*tpdA* mutant strain showed a 1.2-fold change in c-di-GMP levels compared with the WT strain at all time points analyzed. This was the only PDE that contributed to c-di-GMP homeostasis under our experimental conditions in either the WT or Δ*opaR* genetic background (Fig. 2B to F). The Δ*opaR* Δ*tpdA* double mutant showed an intermediate phenotype in terms of c-di-GMP accumulation compared to the single mutants Δ*opaR* and Δ*tpdA* during the first 8 h of growth. It showed higher c-di-GMP levels compared to the Δ*opaR* mutant strain, but lower levels compared to the Δ*tpdA* mutant strain. This result is consistent with the positive regulatory role on c-di-GMP accumulation proposed for OpaR and the negative role proposed for TpdA. This also indicates that the decrease in c-di-GMP accumulation observed in the Δ*opaR* mutant strain is not solely due to an increase in TpdA accumulation. Interestingly, at the 24-h time point, we observed 1.9- and 1.5-fold changes in c-di-GMP levels when comparing the Δ*opaR* Δ*tpdA* mutant strain with the single mutants Δ*opaR* and Δ*tpdA*, respectively (Fig. 2B and F). This result indicates that a later time point, the combined absence of *opaR* and *tpdA* (Δ*opaR* Δ*tpdA*) results in higher c-di-GMP accumulation than that when only *tpdA* is absent. This suggests that when TpdA is absent, OpaR may prevent c-di-GMP accumulation during the late stationary phase of growth in planktonic cultures.

Our observations suggest that TpdA has a stronger influence on c-di-GMP homeostasis than ScrC, ScrG, and VP0117 in planktonic cultures. It remains to be tested whether other OpaR-regulated PDEs or other PDEs in general play equal or major roles compared to TpdA under these growth conditions.

### The upregulation of *tpdA* in the absence of *opaR* requires low levels of c-di-GMP.

**FIG 3:**
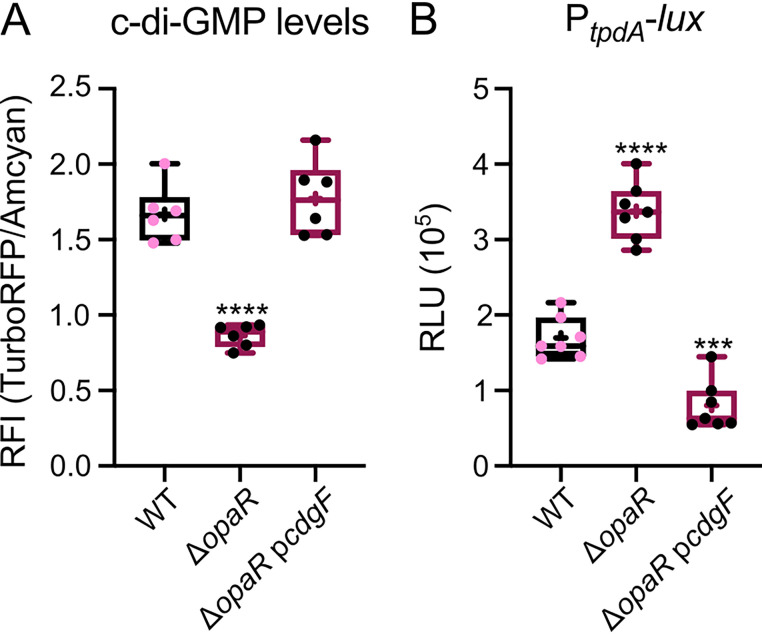
An increase in c-di-GMP levels eliminates the upregulation of *tpdA* observed in the absence of *opaR*. (A) The c-di-GMP levels measured with the c-di-GMP genetic reporter in cells grown for 8 h with agitation at 30°C in the presence of 0.1 mM IPTG (isopropyl-β-d-thiogalactopyranoside). c-di-GMP levels are expressed as RFI values. (B) Expression data, in RLU, of the transcriptional fusion P*_tpdA_*-*luxCDABE* in different genetic backgrounds after 8 h of growth with agitation at 30°C in the presence of 0.1 mM IPTG. Three independent biological samples were analyzed in two separate experiments. Means were compared using a one-way ANOVA followed by a Dunnett’s multiple-comparison test for direct comparison with the mean of the WT strain. Asterisks (*) indicate statistical differences (adjusted *P* values) compared to the WT. ***, *P* ≤ 0.001; ****, *P* ≤ 0.0001.

We have demonstrated that the absence of *opaR* results in a decrease in c-di-GMP accumulation compared to that in the WT strain (Fig. 2) and proposed that this could be the mechanism by which this regulator affects *tpdA* expression. However, OpaR, being a transcriptional regulator, could potentially act directly on the regulatory region of *tpdA*. In fact, some studies have identified potential OpaR binding sites in the regulatory region of *tpdA* (25). Based on these antecedents, we wondered whether the change in c-di-GMP levels in the Δ*opaR* genetic background could be the main cause of the induction of P*_tpdA_* promoter activity. To test this, we artificially elevated the c-di-GMP levels in the Δ*opaR* mutant strain by introducing a plasmid which enables the overexpression of *cdgF* (VCA0956), the product of which is an active DGC from V. cholerae (13). To determine the magnitude of the c-di-GMP increase resulting from *cdgF* overexpression, we introduced a compatible plasmid which has the c-di-GMP biosensor described above (pDZ119) (27). As a control, we introduced the transcriptional fusion or the c-di-GMP biosensor together with the empty expression plasmid into the WT and Δ*opaR* mutant strains to produce conditions under which *cdgF* is not overexpressed. The overproduction of CdgF in the Δ*opaR* mutant strain resulted in c-di-GMP accumulation levels similar to those observed in the WT strain (Fig. 3A). This increase in c-di-GMP levels was enough to eliminate the induction of the P*_tpdA_* promoter (Fig. 3B). This result suggests that upregulation of the P*_tpdA_* promoter in the absence of OpaR requires low levels of c-di-GMP in planktonic cultures. We observed decreased *tpdA* expression in the Δ*opaR*/p*cdgF* genetic background compared to that in the WT/pMMB genetic background. Further analysis is required to determine whether the absence of OpaR negatively affects *tpdA* expression when c-di-GMP levels are artificially elevated.

### The expression of *tpdA* in cells growing on solid medium showed an altered dynamic sensitivity to the absence of OpaR or ScrC compared to that of cells grown in liquid cultures.

**FIG 4:**
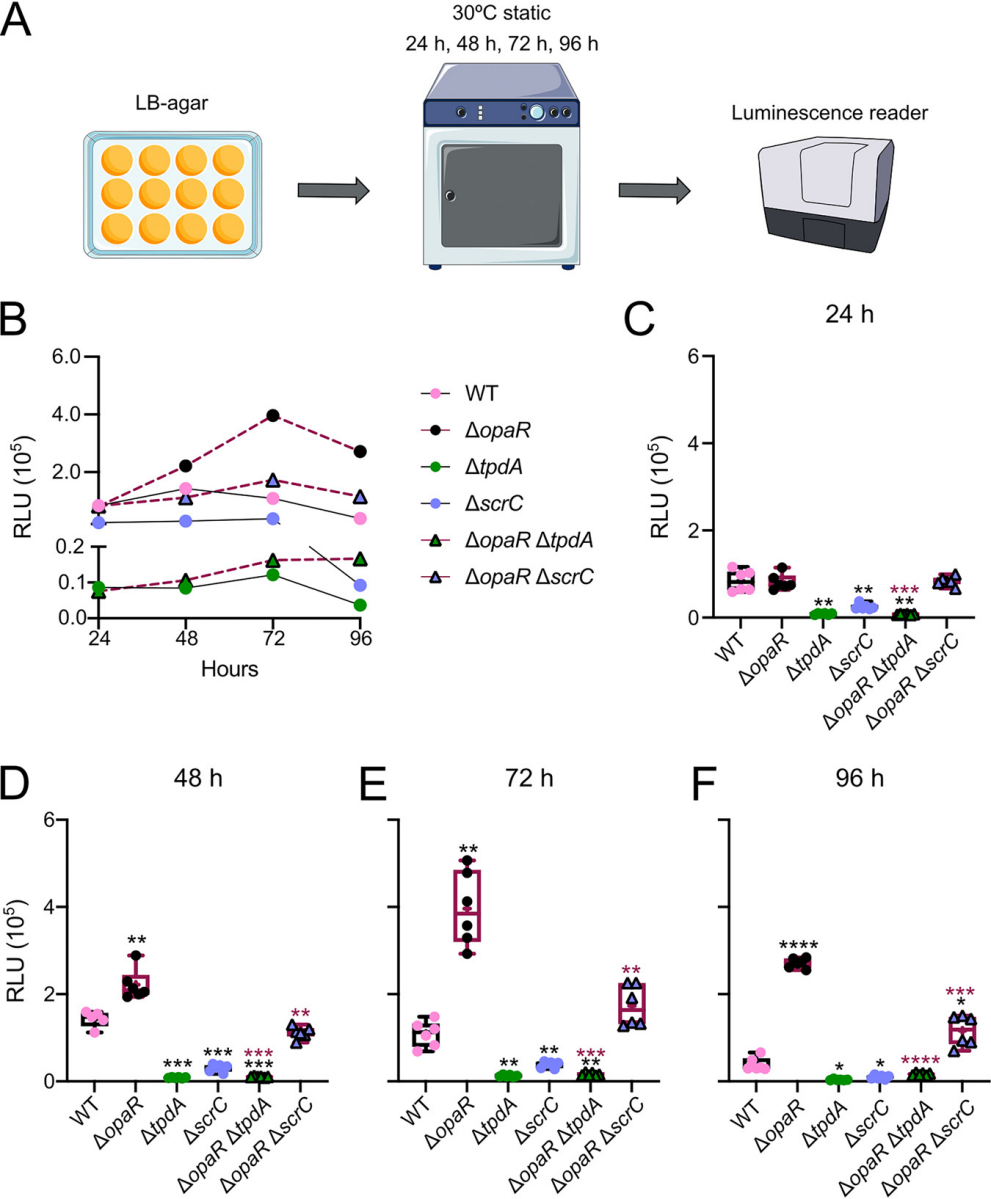
Influence of OpaR on *tpdA* expression increases over time in cells grown on solid medium. (A) Schematic representation of the experimental procedure. Cells were grown statically on LB agar with 5 μg/mL Chl at 30°C. At least five independent biological samples were analyzed across two independent experiments. (B) Graph of the data plotted in panels C to F, showing the mean RLU values for each strain at each time point tested. (C to F) Box plots representing the expression data, in RLU, of the transcriptional fusion P*_tpdA_*-*luxCDABE* in different genetic backgrounds at 4 time points. Means were compared using a Brown-Forsythe and Welch ANOVA followed by a Dunnett’s T3 multiple-comparison test for direct comparison with the mean of either the WT or Δ*opaR* mutant strain. Black and maroon asterisks (*) indicate statistical differences (adjusted *P* values) compared to WT and Δ*opaR*, respectively. *, *P* ≤ 0.05; **, *P* ≤ 0.01; ***, *P* ≤ 0.001; ****, *P* ≤ 0.0001.

Our results so far indicate that OpaR has a negative effect on *tpdA* expression, and that the regulatory mechanism involves the manipulation of c-di-GMP levels partly through the control of ScrC abundance. ScrC production is directly regulated by OpaR, but its activity as a PDE is also influenced by the abundance of an autoinducer-like molecule produced by ScrA (30). It has been speculated that the accumulation of this autoinducer-like signal is favored by a surface-adhered lifestyle (30). These antecedents led us to evaluate the influence of OpaR, ScrC, and TpdA on the transcriptional dynamics of the P*_tpdA_* promoter in cells growing on solid medium (Lysogeny Broth [LB] agar). We analyzed the activity of the transcriptional fusion P*_tpdA_*-*luxCDABE* in the WT, Δ*opaR*, Δ*tpdA*, Δ*scrC*, Δ*opaR* Δ*tpdA*, and Δ*opaR* Δ*scrC* genetic backgrounds in cells grown on LB agar for 24, 48, 72, and 96 h at 30°C (Fig. 4A).

The activity of the P*_tpdA_* promoter in the WT strain reached its peak after 48 h of growth, showing a 1.7-fold change compared to that after 24 h (Fig. 4B). After reaching its peak, the activity of the P*_tpdA_* promoter decreased, showing a 0.5-fold change when comparing that at 24 h of growth to that at 96 h (Fig. 4B to F).

In contrast to what we observed in planktonic cultures, the absence of *opaR* did not affect the activity of the P*_tpdA_* promoter after 24 h of growth, compared to the WT strain (Fig. 4C). However, after this time point, the absence of *opaR* resulted in a gradual increase in P*_tpdA_* promoter activity compared to that in the WT strain, reaching a 7-fold change after 96 h of growth (Fig. 4B to F).

The activity of the P*_tpdA_* promoter in cells growing on LB agar was strongly dependent on the presence of TpdA and ScrC (Fig. 4C to E). The absence of *tpdA* or *scrC* resulted in 0.1- and 0.3-fold changes in activity, respectively, compared to the WT strain after 24 h of growth. These differences in activity were maintained at all time points analyzed. In planktonic cultures, we did not observe an effect on *tpdA* expression in the Δ*scrC* single mutant strain at any time point; hence, it appears that ScrC plays a more determinant role in controlling *tpdA* expression in cells grown on solid medium.

The P*_tpdA_* promoter activity in the Δ*opaR* Δ*scrC* strain was not statistically different compared to that in the WT or Δ*opaR* strains at up to 24 h on LB agar plates (Fig. 4C). From 48 h to 96 h of growth, the P*_tpdA_* activity level in the Δ*opaR* Δ*scrC* strain remained similar to that in the WT strain but decreased compared to that in the Δ*opaR* strain (Fig. 4D to F). The intermediate effect on *tpdA* expression of the combined versus single absence of *opaR* and *scrC* from the 48-h to 96-h time points supports the notion that multiple OpaR-regulated genes are involved in controlling the production of the trigger phosphodiesterase TpdA.

### ScrC and TpdA exchange the dominant role in c-di-GMP degradation after the transition from the early to late growth stages on LB agar, in the absence of OpaR.

**FIG 5:**
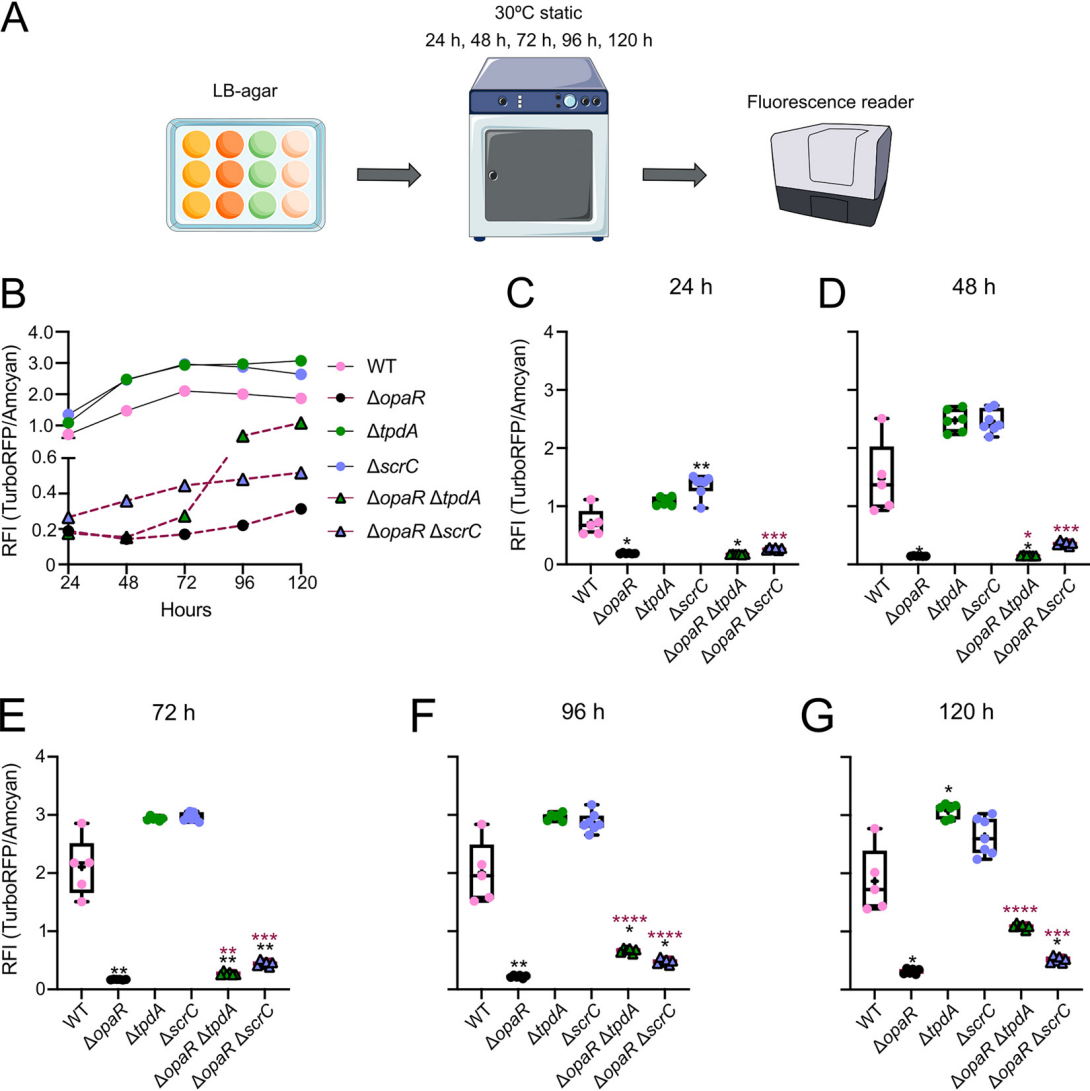
The OpaR-regulated PDEs TpdA and ScrC act at different time windows to control c-di-GMP metabolism in cells growing on solid medium. (A) Schematic representation of the experimental procedure. Cells were grown statically on LB agar with 15 μg/mL Gen at 30°C. At least 5 independent biological samples were analyzed across three independent experiments. (B) Graph of the data plotted in panels C to G, showing the mean RFI values for each strain at each time point tested. (C to G) Box plots representing RFI values, which are proportional to the levels of c-di-GMP in the colonies at 5 different time points. Means were compared using a Brown-Forsythe and Welch ANOVA followed by a Dunnett’s T3 multiple-comparison test for direct comparison with the mean of either the WT strain or the Δ*opaR* mutant strain. Black and maroon asterisks (*) indicate statistical differences (adjusted *P* values) compared to WT and Δ*opaR*, respectively. *, *P* ≤ 0.05; **, *P* ≤ 0.01; ***, *P* ≤ 0.001; ****, *P* ≤ 0.0001.

Our observations regarding the P*_tpdA_* promoter activity in cells grown on LB agar prompted us to analyze the dynamics of c-di-GMP accumulation under the same growth conditions (Fig. 5A). In the WT strain, c-di-GMP levels reached a peak after 72 h of growth, showing a 3-fold change compared to that at 24 h (Fig. 5B). The absence of *opaR* resulted in a 0.1-fold change in c-di-GMP levels, during the peak of accumulation of this second messenger, compared to that in the WT strain (Fig. 5B to E). This strongly suggests that OpaR plays a key role in promoting c-di-GMP accumulation in cells growing on LB agar. We previously showed that *tpdA* expression is not altered in the Δ*opaR* mutant strain compared to the WT strain after 24 h of growth on LB agar (Fig. 4B), yet we observed a 0.3-fold change in c-di-GMP levels when comparing the Δ*opaR* mutant strain and the WT strain at the 24-h time point. This leads us to propose that there are factors other than c-di-GMP levels which influence P*_tpdA_* promoter activity at early time points during the growth of cells on LB agar.

The differences in c-di-GMP accumulation between the WT strain and the *scrC* and *tpdA* single mutants were more evident at the 24- and 120-h time points, respectively (Fig. 5B, C, and G). After 24 h of growth, we observed a 1.9-fold change in c-di-GMP accumulation in the Δ*scrC* mutant strain compared to the WT strain, while after 120 h of growth, we observed a 1.6-fold change in c-di-GMP levels in the Δ*tpdA* mutant strain compared to the WT strain. The involvement of TpdA and ScrC in controlling c-di-GMP homeostasis in cells growing on LB agar was more evident when comparing c-di-GMP levels between the Δ*opaR* single mutant and the double mutants Δ*opaR* Δ*tpdA* and Δ*opaR* Δ*scrC.* The c-di-GMP levels in the Δ*opaR* and Δ*opaR* Δ*tpdA* mutant strains were very similar after 24 and 48 h of growth (Fig. 5B, C, and D). However, after the 72-h time point, c-di-GMP levels started to increase in the Δ*opaR* Δ*tpdA* mutant strain compared to those in the Δ*opaR* mutant strain, reaching a 3.5-fold change after 120 h of growth (Fig. 5B and Fig. 5E to G). This result suggests that TpdA does not play a dominant role in depleting c-di-GMP levels in the absence of OpaR at the early time points (24 and 48 h), but its presence is very influential in controlling c-di-GMP accumulation at later time points.

In contrast to what we observed in the absence of *tpdA*, the absence of *scrC* in the Δ*opaR* genetic background (Δ*opaR* Δ*scrC*) influenced c-di-GMP accumulation at early time points (between 24 and 72 h of growth), showing up to a 2.6-fold change compared to that in the Δ*opaR* mutant strain (Fig. 5B and Fig. 5C to E). However, at 96 and 120 h of growth, the increase in c-di-GMP levels was higher in the Δ*opaR* Δ*tpdA* mutant strain compared to that in the Δ*opaR* Δ*scrC* mutant strain, suggesting that the dominant influence in controlling c-di-GMP levels in cells growing on LB agar in the absence of OpaR switches from ScrC to TpdA around the 96-h time point (Fig. 5B, E, and F).

In summary, our data suggest that the OpaR-regulated PDEs ScrC and TpdA act during different stages of colony growth to control c-di-GMP levels. The different influences of ScrC and TpdA on c-di-GMP homeostasis are more evident in the absence of OpaR, perhaps due to the negative effect of this regulator on both *scrC* and *tpdA* expression. It is also clear from these results that OpaR can control c-di-GMP accumulation in cells grown on LB agar through additional regulators besides ScrC and TpdA.

### OpaR, ScrC, and TpdA affect the dynamics of *cpsA* expression in cells growing on LB agar.

**FIG 6:**
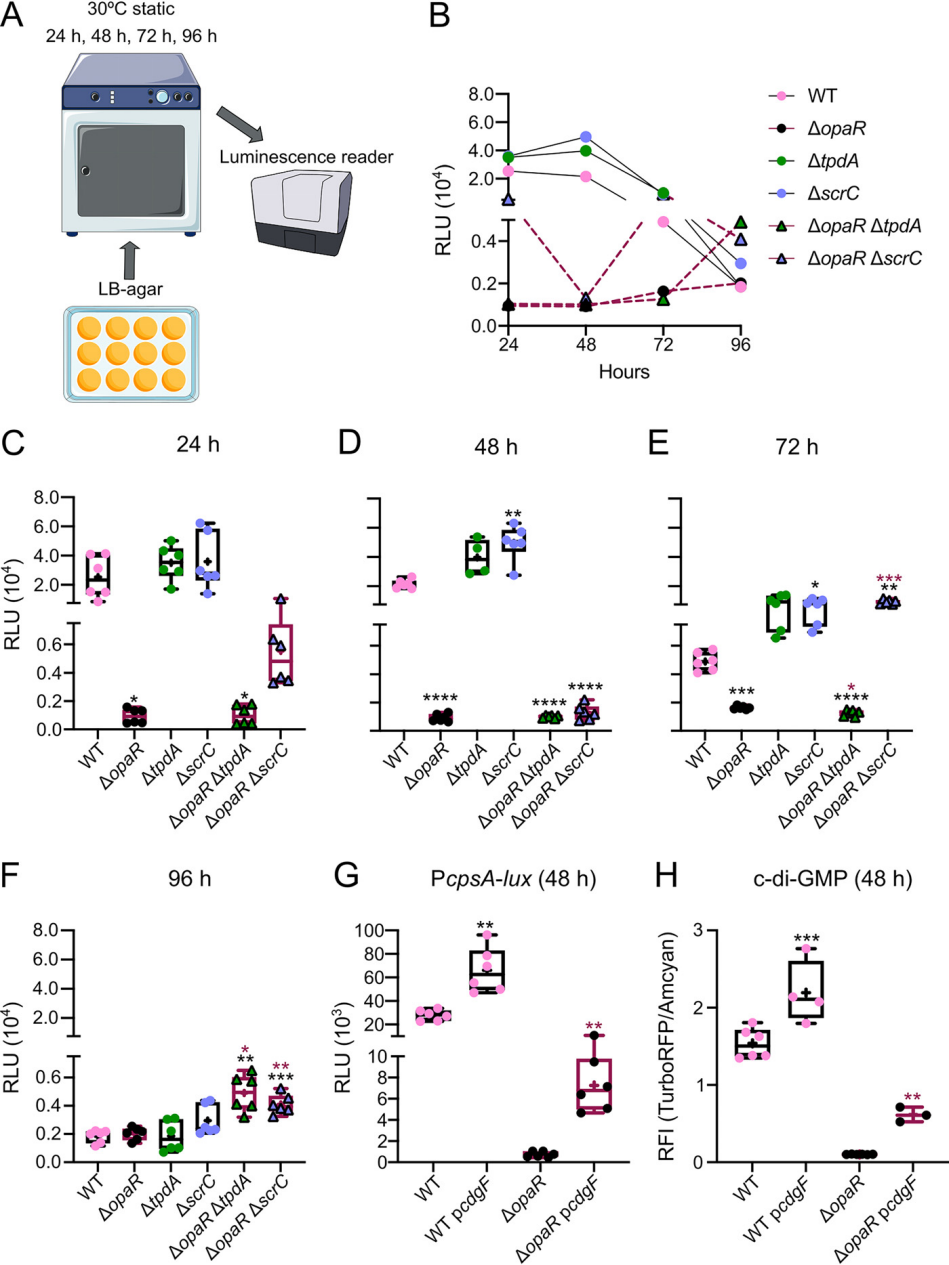
OpaR and its regulated PDEs contribute to control *cpsA* expression over time in cells grown on solid medium. (A) Schematic representation of the experimental procedure. Cells were grown statically over LB agar with 5 μg/mL Chl at 30°C. At least 4 independent biological samples were analyzed across two independent experiments. (B) Graph of the data plotted in panels C to F showing the mean RLU values for each strain at each time point tested. (C to G) Box plots representing the expression data, in RLU, of the transcriptional fusion P*_cpsA_*-*luxCDABE* in different genetic backgrounds at different time points. (H) Box plot representing fluorescence data, in RFI, of cells with different genetic backgrounds expressing the c-di-GMP biosensor after 48 h of growth on LB agar. Means were compared using a Brown-Forsythe and Welch ANOVA followed by a Dunnett’s T3 multiple-comparison test for direct comparison with the mean of either the WT or Δ*opaR* mutant strain. Black and maroon asterisks (*) indicate statistical differences (adjusted *P* values) compared to WT and Δ*opaR*, respectively. *, *P* ≤ 0.05; **, *P* ≤ 0.01; ***, *P* ≤ 0.001; ****, *P* ≤ 0.0001.

The expression of *cpsA* is positively regulated by c-di-GMP abundance through a hierarchical array of regulators (27, 31, 34, 35). We analyzed whether the drop or elevation in c-di-GMP levels experienced by our strains of interest when grown on LB agar results in altered expression of *cpsA* (Fig. 6A). To analyze *cpsA* expression, we used a previously reported transcriptional fusion with the P*_cpsA_* promoter fused to the reporter genes *luxCDABE* (27). Our results revealed that *cpsA* expression is negatively affected by the absence of OpaR at all time points analyzed compared to that in the WT strain (Fig. 6B to F) and is positively impacted by the absence of ScrC after 48 and 72 h of growth (Fig. 6B, D, and E). This correlates with the positive and negative effects of OpaR and ScrC, respectively, on c-di-GMP accumulation. The expression of *cpsA* in the Δ*opaR* Δ*tpdA* mutant strain showed similar levels to those observed in the Δ*opaR* mutant strain from the 24- to the 72-h time point (Fig. 6B, Fig. 6C to E). It is important to remember that at these time points, ScrC is more influential than TpdA in lowering c-di-GMP levels in the Δ*opaR* background, and that this dynamic changes after 96 h of growth. The expression of *cpsA* in the Δ*opaR* Δ*tpdA* mutant strain showed approximately a 2.5-fold change compared to the WT strain and the single mutants at the 96-h time point (Fig. 6B and F). This is another instance where we observed that the combined absence of *tpdA* and *opaR* had a cumulative effect, in this case over the induction of the P*_cpsA_* promoter. Here, we also observed a 2-fold change in the activity of the P*_cpsA_* promoter when comparing the Δ*opaR* Δ*scrC* mutant strain with the other strains analyzed (Fig. 6B and F). Based on this, we propose that in the absence of TpdA or ScrC, OpaR can negatively affect the activity of the P*_cpsA_* promoter during later stages of growth on LB agar.

We further analyzed whether the reduction in *cpsA* expression in the Δ*opaR* genetic background could be alleviated by artificially increasing the level of c-di-GMP. To do this, we used the same plasmid as shown in Fig. 3 to overexpress *cdgF* and promote c-di-GMP accumulation. The overexpression of *cdgF* resulted in a 1.4-fold change in c-di-GMP levels and a 2.3-fold change in *cpsA* expression in the WT strain (WT/empty plasmid versus WT/p*cdgF*) (Fig. 6G and H). The overexpression of *cdgF* in the Δ*opaR* mutant strain did not enable it to reach WT levels of c-di-GMP (Fig. 6G and H). However, it promoted a 6-fold change in c-di-GMP accumulation compared to that in a Δ*opaR* genetic background that does not overexpress *cdgF* (Fig. 6G and H). The elevation in c-di-GMP levels in the Δ*opaR* genetic background overexpressing *cdgF* resulted in a 10-fold change in *cpsA* expression compared to the Δ*opaR* genetic background lacking *cdgF* (Fig. 6G and H).

Together, our data suggest that OpaR positively regulates *cpsA* expression in cells growing on solid medium. It appears that the positive effect of OpaR on *cpsA* expression, under these growth conditions, is at least partially influenced by its role as a positive modulator of c-di-GMP accumulation. However, we cannot rule out the possibility that OpaR directly activates the transcription of *cpsA*. During later growth stages, if c-di-GMP levels are elevated due to the absence of *tpdA* or *scrC*, the absence of *opaR* results in increased *cpsA* expression, suggesting that OpaR may also act as a negative regulator of this gene under certain conditions.

### OpaR can inhibit biofilm formation and *cpsA* expression.

**FIG 7:**
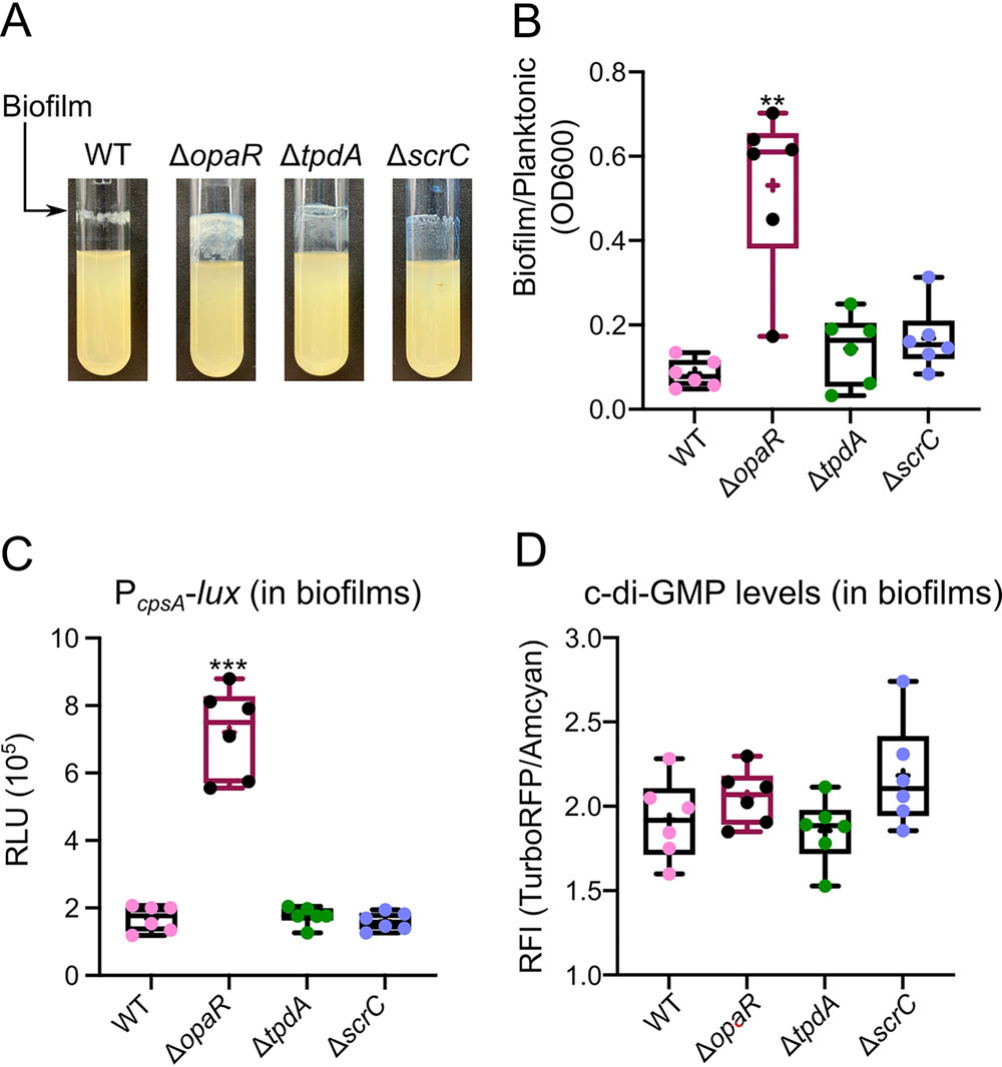
OpaR limits biofilm formation and *cpsA* expression in biofilms grown on glass with agitation. (A) Representative pictures of 24-h biofilms grown under shaking conditions over the glass surfaces of test tubes at 30°C. (B) Box plots representing the quantification of biofilm formation over glass under shaking conditions. (C) Box plots representing the expression data, in RLU, of the transcriptional fusion P*_cpsA_*-*luxCDABE* in different genetic backgrounds. Biofilms expressing P*_cpsA_*-*luxCDABE* were harvested after 24 h of growth in agitation at 30°C. (D) Box plots representing the c-di-GMP levels, expressed as RFI values, measured with the c-di-GMP genetic reporter in biofilms grown with agitation at 30°C for 24 h. Means were compared using a Brown-Forsythe and Welch ANOVA followed by a Dunnett’s T3 multiple-comparison test for direct comparison with the mean of the WT strain. Asterisks (*) indicate statistical differences (adjusted *P* values) compared to WT. **, *P* ≤ 0.01; ***, *P* ≤ 0.001.

Our results so far support the role of OpaR as primarily a positive regulator of *cpsA* expression, and consequently, of biofilm matrix production in cells growing on solid medium, but also suggest a potential secondary role as a negative regulator of *cpsA* under certain conditions (20, 21, 24, 25, 36). When growing overnight cultures of the Δ*opaR* mutant strain under shaking conditions, we noticed the formation of a biofilm on the surfaces of glass test tubes that was thicker than the biofilm formed by the WT strain (Fig. 7A and B). Thus, we decided to quantitatively analyze the levels of *cpsA* expression and c-di-GMP accumulation in this type of biofilm to see whether the negative regulatory role of OpaR was more evident under these growth conditions.

First, we quantified the amount of biofilm formed by the WT, Δ*opaR* mutant, Δ*tpdA* mutant, and Δ*scrC* mutant strains in cultures grown under shaking conditions. The Δ*opaR* mutant strain showed a 6-fold change in biofilm formation compared to the WT strain, while the *tpdA* and *scrC* null mutants produced similar levels of biofilm to the WT strain (Fig. 7A and B).

Next we used strains harboring either the plasmid pDZ46 or the plasmid pFY4535, which have the P*_cpsA_*-*luxCDABE* transcriptional fusion and the c-di-GMP biosensor, respectively, to analyze *cpsA* expression and c-di-GMP levels in biofilms. We observed a 4-fold change when comparing P*_cpsA_* promoter activity in the Δ*opaR* mutant strain to that in the WT strain (Fig. 7C). The levels of *cpsA* expression in the Δ*tpdA* and Δ*scrC* mutant strains were not different from that observed in the WT strain (Fig. 7C). This would suggest that under the growth conditions and time points analyzed, *cpsA* expression is strongly inhibited by OpaR but not by ScrC or TpdA. We did not observe differences in c-di-GMP accumulation in biofilms formed by any of the strains analyzed (Fig. 7D). This suggests that the absence of *opaR* does not negatively impact c-di-GMP accumulation in these type of biofilms; however, further studies are required to support this observation.

**FIG 8:**
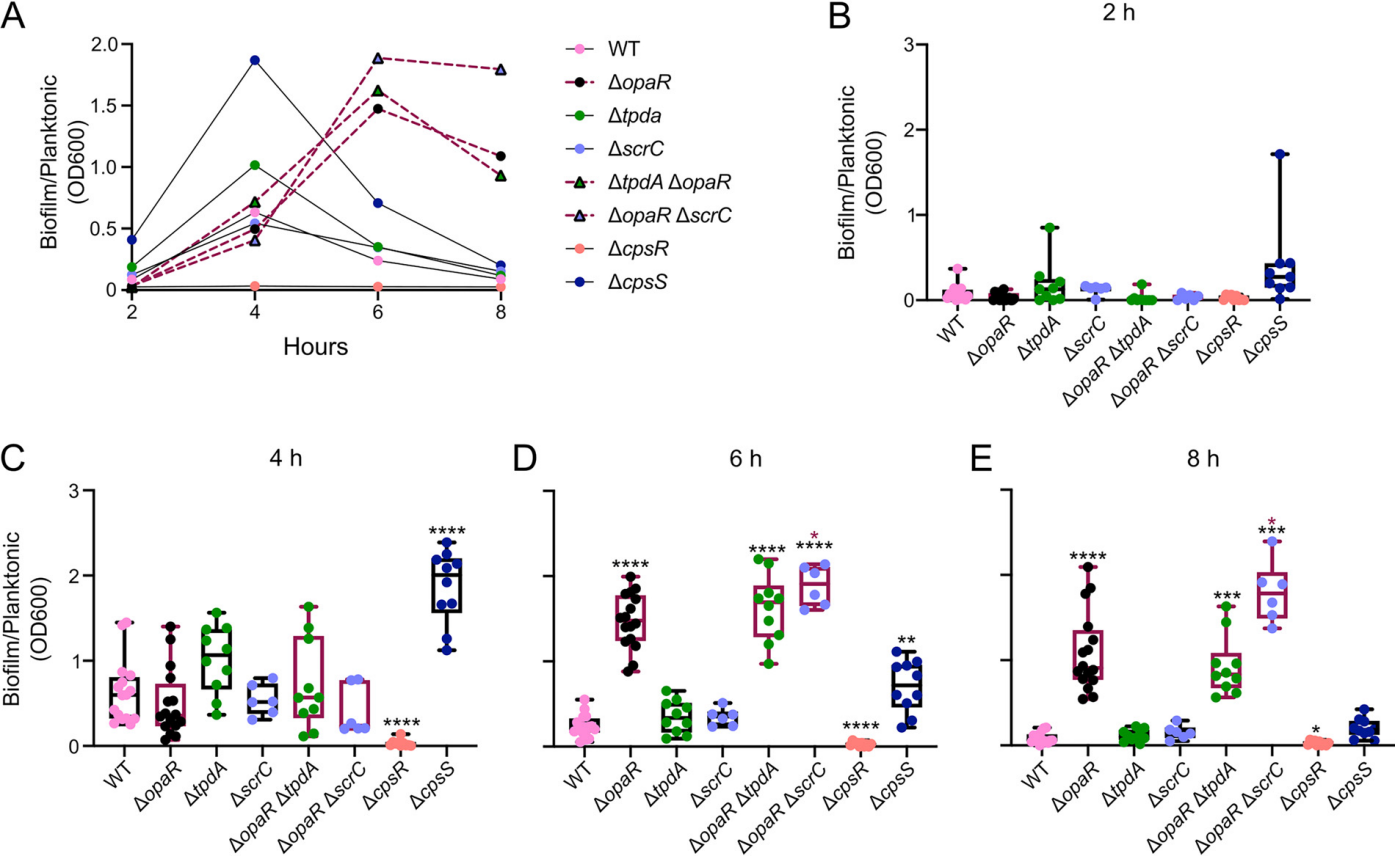
OpaR plays a key role in controlling biofilm formation kinetics on a PVC surface. (A) Graph of the data plotted in panels B to E, showing the mean values of biofilm formation for each strain at each time point tested. (B to E) Quantification of biofilm formation on a PVC surface for strains of interest under static conditions at 30°C. Biofilm formation values are the proportion between the OD_600_ of biofilms stained with crystal violet and the OD_600_ of the planktonic culture. (E) Means were compared using a Brown-Forsythe and Welch ANOVA followed by a Dunnett’s T3 multiple-comparison test for direct comparison with the mean of the WT or Δ*opaR* mutant strain. Black and maroon asterisks (*) indicate statistical differences (adjusted *P* values) compared to WT and Δ*opaR*, respectively. *, *P* ≤ 0.05; **, *P* ≤ 0.01; ***, *P* ≤ 0.001; ****, *P* ≤ 0.0001.

We next evaluated whether under our experimental conditions, the Δ*opaR* mutant strain had an altered biofilm phenotype compared to the WT strain under static growth on a plastic surface (PVC), as previously reported in the genetic background of strain BB22 (24). We analyzed the kinetics of biofilm formation under static conditions in PVC wells of the WT strain, the Δ*opaR*, Δ*tpdA*, Δ*scrC*, Δ*opaR* Δ*tpdA*, and Δ*opaR* Δ*scrC* mutant strains, and control strains with low (Δ*cpsR*) or high biofilm formation capacity (Δ*cpsS*) (Fig. 8) (21). The WT strain showed a peak in biofilm formation after 4 h of growth on LB at 30°C; after this time point, the biofilm started to disperse and/or detach (Fig. 8A). The Δ*opaR* mutant strain showed similar levels of biofilm formation compared to the WT strain after 4 h of growth (Fig. 8C). However, at the 6-h time point, the Δ*opaR* mutant strain showed a 6-fold change in biofilm formation compared to the WT strain (Fig. 8D). The 6-h time point marked the peak of biofilm formation for the Δ*opaR* mutant strain (Fig. 8A). In the WT strain, biofilm formation decreased after 6 and 8 h of growth compared to that at the 4-h time point; while in the Δ*opaR* mutant strain, biofilm formation remained stable from the 6- to 8-h time points (Fig. 8A). It can be proposed that the absence of *opaR* partially prevents biofilm dispersal and/or detachment, perhaps by extending the time window of the expression of biofilm-related genes. These results are similar to the previously reported observations of the V. parahaemolyticus strain BB22 (24).

The individual absence of *tpdA* or *scrC* does not affect biofilm formation compared to that in the WT strain. When *tpdA* was eliminated together with *opaR*, the biofilm-formation phenotype resembled that of the Δ*opaR* mutant strain. When *scrC* was eliminated together with *opaR*, we observed an increase in biofilm formation compared to the Δ*opaR* mutant strain. These results suggest that TpdA does not play a major role in controlling biofilm formation under the conditions tested, while the role of ScrC in biofilm formation becomes apparent when its negative regulator OpaR is absent.

The Δ*cpsR* mutant showed a severe defect in biofilm formation, which is expected based on the role of CpsR as a positive regulator of *cps* genes (Fig. 8) (21). The Δ*cpsS* mutant strain showed a 3-fold change in biofilm formation at the 4-h time point compared to the WT strain. This result is to be expected since it has been reported that CpsS represses *cpsR* expression (21). The biofilm formed by the Δ*cpsS* mutant was almost completely dispersed and/or detached after 8 h of growth. The differences in the biofilm formation kinetics of the Δ*opaR* and Δ*cpsS* mutant strains could suggest that these regulators repress biofilm formation through different mechanisms and/or during different stages of biofilm development.

### OpaR interacts with the regulatory region of a variety of genes involved in c-di-GMP metabolism and biofilm formation.

**FIG 9:**
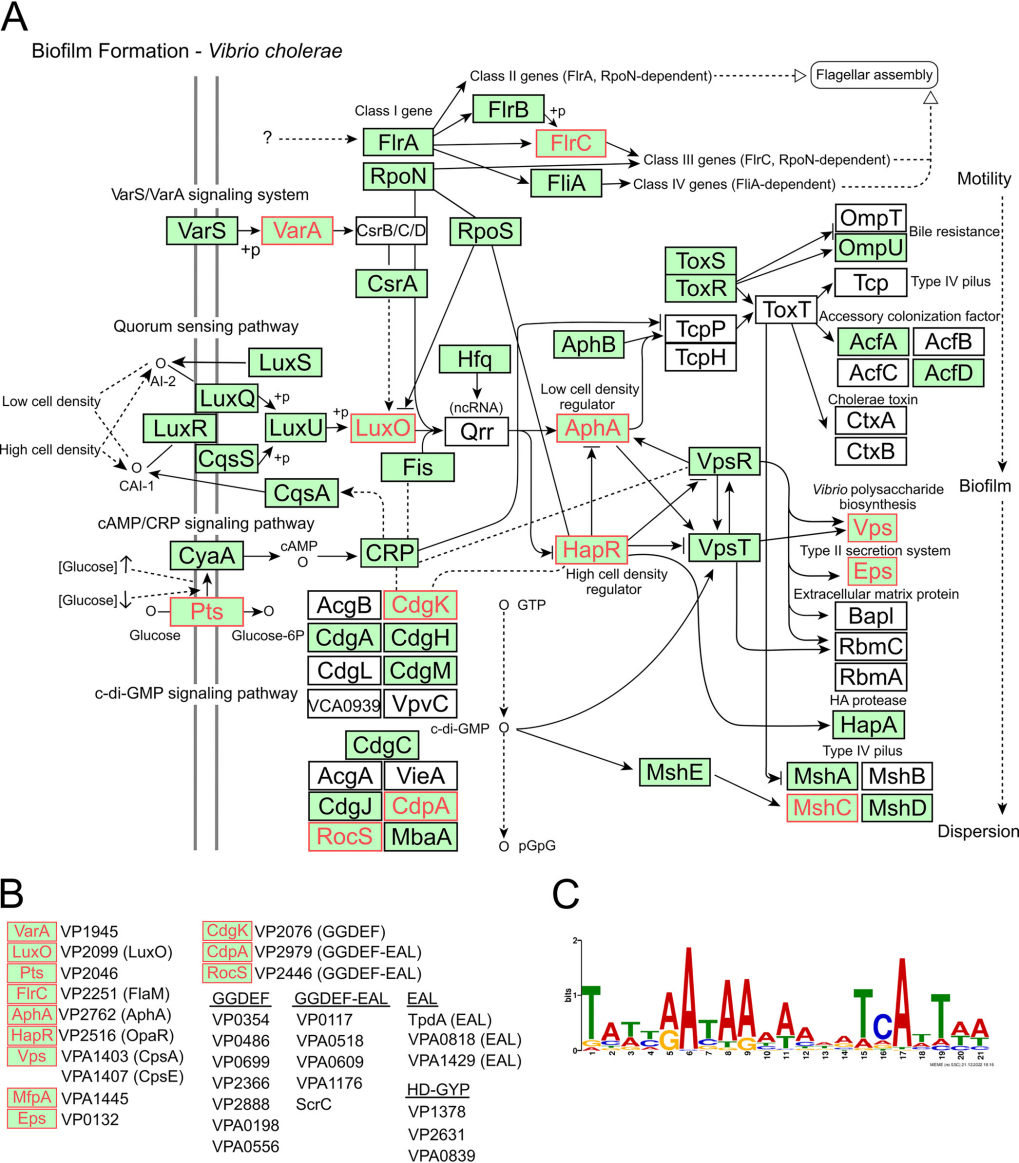
OpaR has potential regulatory roles at several branches of the biofilm regulatory circuit. (A) Schematic representation of the biofilm formation pathway generated with KEGG Mapper to illustrate gene products that are conserved in the V. parahaemolyticus RIMD2210633 and genes retrieved from a previously reported chromatin immunoprecipitation sequencing (ChIP-seq) experiment focused on elucidating OpaR-DNA interactions during the late-exponential and stationary growth phases (GEO accession no. GSE122479). Notations are as described in the documentation for KEGG Pathways Map. Green boxes indicate gene products which, based on the KEGG database, are also present in V. parahaemolyticus. Gene products labeled in red were retrieved from the analysis of raw data from the ChIP-seq experiment mentioned above. Filled arrows indicate positive regulation or interaction; T connectors indicate negative regulation or inhibition. Dashed lines indicate indirect interaction or an uncharacterized association. Unfilled arrows indicate a connection with another pathway map from the KEGG database. (B) Biofilm and c-di-GMP metabolism-related gene products from V. parahaemolyticus obtained from the analysis of ChIP-seq data for OpaR-DNA interactions. (C) Logo of the conserved motif identified within the peaks obtained from the ChIP-seq data for OpaR-DNA interactions associated with biofilm- and c-di-GMP-related genes. The Multiple Em for Motif Elicitation (MEME) program was used to generate the logo.

The genome-wide landscape of OpaR-DNA interactions during the exponential and stationary growth phases was recently published and the data uploaded to the Gene Expression Omnibus (GEO) data set under accession no. GSE122479 (37). We utilized the raw data of a chromatin immunoprecipitation sequencing (ChIP-seq) experiment to identify DNA regions recognized by OpaR. We then focused our attention on genes associated with biofilm formation and c-di-GMP metabolism. For this purpose, we used the tool KEGG Mapper to search for the presence of mapped objects in the V. cholerae biofilm formation pathway in the data set of genes recognized by OpaR (Fig. 9). This data set contained gene products involved in motility, biofilm matrix production, adherence to surfaces, and biofilm dispersion. *opaR* and *aphA* were part of the data set of genes recognized by OpaR, supporting a previous observation of their direct regulation by this protein (38). The OpaR-ChIP-seq data set also contained the gene *luxO* (VP2099), whose product indirectly regulates OpaR accumulation (25), and the gene VP1945, whose product is an orthologue of VarA from V. cholerae. VarA is a response regulator that controls the abundance of the sRNA binding protein CsrA, which indirectly modulates LuxO activity (39) and directly regulates AphA expression (40). We also identified 21 genes in the ChIP-seq data set whose products are predicted or known to be involved in c-di-GMP metabolism (Fig. 9B). Of these, three are objects in the biofilm formation pathway of V. cholerae: the DGC CdgK and the PDEs CdpA and RocS (14, 41, 42). The ChIP-seq data set also contained c-di-GMP related genes previously shown to be directly regulated by OpaR (26) and the gene *tpdA*. Our data suggest that OpaR regulates *tpdA* expression mainly through controlling c-di-GMP levels; however, the ChIP-seq data could support an additional regulatory mechanism involving direct transcriptional regulation.

We were able to identify a conserved OpaR binding sequence (Fig. 9C), similar to the one reported in the original work which generated the ChIP-seq data set (37), in all the genes mapped to the biofilm formation pathway of V. cholerae and the c-di-GMP related genes shown in Fig. 9B. The direct involvement of OpaR in the transcriptional regulation of *tpdA* and the multiple c-di-GMP-related genes found within the ChIP-seq database deserves further attention and could give us a bigger picture of the regulatory circuitry that can be exploited by modulating OpaR abundance.

## DISCUSSION

c-di-GMP accumulation in V. parahaemolyticus plays a key role in the adaptation to a surface-adhering lifestyle and surface colonization, not only during biofilm formation but also during the process of swarming motility, which involves the movement over solid or semisolid surfaces using lateral flagella (29, 30, 43). Our knowledge of the mechanisms which govern changes in c-di-GMP accumulation in V. parahaemolyticus is limited compared to what has been reported for V. cholerae (12). Nonetheless, several PDEs which are present in V. parahaemolyticus but absent in V. cholerae have been reported to play crucial roles in surface sensing, biofilm formation, and swarming motility (27, 29–31). ScrC is the best characterized of these PDEs; in fact, ScrC is a dual-function enzyme capable of c-di-GMP synthesis and degradation. The switch-in activity of ScrC is regulated through its interaction with ScrB, a periplasmic protein capable of binding a quorum-like autoinducer molecule named Signal S, produced by ScrA. ScrA, ScrB, and ScrC are encoded in an operon located in the small chromosome of V. parahaemolyticus (VPA1513/*scrA*-VPA1512/*scrB*-VPA1511/*scrC*). The expression of this operon is crucial for the process of swarming motility and is negatively regulated by OpaR (25, 29). The PDE ScrG is also capable of regulating cellular behaviors on solid or semisolid medium, and its expression has been reported to be positively regulated by OpaR (26, 31). Finally, the trigger phosphodiesterase TpdA, one of the main subjects of study in this work, has been previously shown to positively modulate swimming motility and, when overproduced, to inhibit biofilm formation and promote swarming motility (27). In that study, we reported that the activity of the P*_tpdA_* promoter is induced by a decrease in c-di-GMP levels, but the magnitude of change in c-di-GMP necessary for this induction was not investigated. Although the three previously mentioned PDEs affect phenotypes related to surface adaptation, little is known about the subtleties of their roles in controlling the c-di-GMP global pool. The OpaR-regulated PDEs ScrC, ScrG, and VP0117 showed different degrees of contribution to the upregulation of *tpdA* in the absence of OpaR. Although VP0117 was more influential than ScrG in controlling *tpdA* expression in the absence of *opaR*, its contribution was minor compared to the regulation exerted by ScrC. VP0117 is a GGDEF-EAL-domain containing protein whose predominant enzymatic activity has not yet been characterized, but based on these results it would appear to act as a PDE. We selected VP0117 as a potentially influential PDE in the control of c-di-GMP degradation by OpaR because it was reported to be one of the most highly expressed and induced c-di-GMP metabolizing enzymes in the absence of OpaR (26). Interestingly, the regulatory role of ScrC, ScrG, and VP0117 over *tpdA* in the absence of OpaR was not accompanied by significant changes in c-di-GMP accumulation in cells growing in liquid cultures. This suggests that the activity of the P*_tpdA_* promoter can respond to the absence of particular PDEs without major changes in the global c-di-GMP pool. We also show that the contribution of TpdA and ScrC to the control of c-di-GMP homeostasis depends on the growth conditions: in shaking cultures, TpdA plays a major role compared to ScrC, while in cells growing on solid medium, their roles are swapped during the early stages of growth, but TpdA later regains its dominant influence.

OpaR has been previously shown to positively control c-di-GMP accumulation, a role which fits well with its ability to positively control CPS production and negatively control swarming motility (20, 25). However, it has also been shown that the absence of *opaR* inhibits biofilm dispersion and/or detachment in strain BB22 and promotes biofilm formation in strain RIMD2210633 (24, 26), outcomes that would be typically associated with an elevation in c-di-GMP levels (9). These antecedents suggest that OpaR can both positively and negatively regulate c-di-GMP accumulation, *cpsA* expression, and biofilm formation. Is this dichotomy due to strain differences or a response to as-yet poorly understood environmental factors that can favor one OpaR regulatory role over the other? Although we cannot rule out differences arising from strain variability, our data strongly suggest that OpaR has this yin-and-yang regulatory nature in the strain RIMD2210633. Which side of the double-edged sword OpaR uses to control c-di-GMP metabolism and *cpsA* expression likely depends on growth conditions such as planktonic versus surface-attached, low or high population density, and/or nutrient availability or the accumulation of metabolic byproducts during later stages of growth. OpaR abundance is regulated by changes in the accumulation of quorum-sensing cues (25). The perception of these cues can be affected by factors such as diffusion rates or a lifestyle that involves a semipermeable enclosure typical of biofilms (44–46). Although we did not directly analyze changes in OpaR abundance under our experimental conditions, it is possible that this plays a role in which outlets of the OpaR regulon are altered and how they affect multicellular behaviors.

Our data suggest that TpdA and ScrC are important modulators of c-di-GMP metabolism within the regulatory circuitry of OpaR. Besides being regulated by OpaR, other temporal and functional sequestration strategies appear to control their influence in maintaining c-di-GMP homeostasis in cells growing on solid medium. Temporal and functional sequestration modes of control for c-di-GMP modules refer to strategies that regulate the time for production of c-di-GMP metabolizing enzymes or associated effectors, or mechanisms that allosterically control the enzymatic activity of PDEs and DGCs (11). One result that caught our attention was that although c-di-GMP levels were drastically reduced after 24 h of growth on solid medium in cells that lacked *opaR*, the expression of *tpdA* was not induced. In contrast, in planktonic cultures, a smaller decrease in c-di-GMP levels in cells lacking *opaR* was enough to induce P*_tpdA_* promoter activity, even in shorter periods. This suggests that other regulators besides OpaR and TpdA might control P*_tpdA_* promoter expression in surface-attached cells. The identity of these regulators and their mechanisms of action will be explored in the future.

In V. cholerae, HapR, the orthologue of OpaR, has been shown to regulate multiple c-di-GMP metabolizing enzymes, both PDEs and DGCs (17, 23). So far, the absence of HapR has only been associated with an elevation in c-di-GMP levels (14, 17, 19). This suggests that it negatively regulates the abundance of DGCs and positively regulates the abundance of PDEs, although this does not seem to be as straightforward (17, 23, 47). Based on the *in-silico* analysis of data obtained from a previously reported ChIP-seq experiment focused on OpaR-DNA interactions (37), we identified multiple DGCs, PDEs, and potential dual-function GGDEF-EAL proteins which may be regulated by OpaR. Several of these regulatory relationships have already been explored. At least two potential DGCs, VPA0198 and VP0699, have been shown to be negatively regulated by OpaR. There are at least 5 additional proteins which harbor a GGDEF domain that could be regulated by OpaR. This regulatory relationship and their enzymatic activity require further investigation to determine whether all or some of them contribute to the control of c-di-GMP homeostasis by OpaR. In addition to potential targets involved in c-di-GMP metabolism, targets involved in polar flagellum assembly and surface attachment, such as *flaM* and *mshC*, could begin to explain the biofilm-dispersion phenotype associated with the absence of OpaR. Based on our results, we propose that functional genomic studies of the role of OpaR on c-di-GMP synthesis and degradation and on multicellular behaviors need to be designed considering the dynamic nature of the OpaR-mediated regulation of these processes.

## MATERIALS AND METHODS

### Strains, plasmids, and growth conditions.

The strains and plasmids used in this study are described in Table 1. All strains were grown in Lysogeny Broth (LB) (1% tryptone, 0.5% yeast extract, and 1% NaCl) or on LB agar (LB with 1.5% bacteriological agar). Strains derived from V. parahaemolyticus RIMD2210633 were grown at 30°C, and Escherichia coli strains were grown at 37°C. Strains were grown on solid medium under static conditions and in LB broth under shaking conditions at 200 rpm, at the previously described temperatures. Strains and plasmids were selected by adding antibiotics to the growth media at the following concentrations: streptomycin (Str) at 200 μg/mL, kanamycin (Kan) at 30 μg/mL, gentamicin (Gen) at 15 μg/mL, and chloramphenicol (Chl) at 20 μg/mL for E. coli and 5 μg/mL for V. parahaemolyticus.

**TABLE 1:** Strains and plasmids used in this study

Strain or plasmid	Relevant genotype	Source or reference
E. coli strain		
SY327 (λ*pir*)	Δ(*lac pro*) *argE*(Am) *rif nalA recA56* λ*pir*	
SM10 (λ*pir*)	*Thi, thr, leu, tonA, lacY, supE, recA,* RP4-2-Tc::Mu λ*pir* Kmr	55
V. parahaemolyticus strains		
DZ_Vp_42	RIMD2210633, St^r^, Ap^r^	56, 57
DZ_Vp_113	DZ_Vp_42, Δ*tpdA*, St^r^, Ap^r^	27
DZ_Vp_209	DZ_Vp_42, Δ*opaR*, St^r^, Ap^r^	This work
DZ_Vp_433	DZ_Vp_42, Δ*scrC*, St^r^, Ap^r^	This work
DZ_Vp_444	DZ_Vp_42, Δ*scrG*, St^r^, Ap^r^	This work
DZ_Vp_468	DZ_Vp_42, ΔVP0117, St^r^, Ap^r^	This work
DZ_Vp_382	DZ_Vp_42, Δ*opaR* Δ*tpdA*, St^r^, Ap^r^	This work
DZ_Vp_435	DZ_Vp_42, Δ*opaR* Δ*scrC*, St^r^, Ap^r^	This work
DZ_Vp_447	DZ_Vp_42, Δ*opaR* Δ*scrG*, St^r^, Ap^r^	This work
DZ_Vp_472	DZ_Vp_42, Δ*opaR* ΔVP0117, St^r^, Ap^r^	This work
DZ_Vp_157	DZ_Vp_42, Δ*cpsR*, St^r^, Ap^r^	This work
DZ_Vp_179	DZ_Vp_42, Δ*cpsS*, St^r^, Ap^r^	This work
Plasmids		
pDZ_63 (pRE118)	*oriT oriV sacB aphA*, Km^r^	58
pDZ_118	pRE118-*tpdA*del, Km^r^	27
pDZ_179	pRE118-*opaR*del, Km^r^	This work
pDZ_366	pRE118-*scrC*del, Km^r^	This work
pDZ_364	pRE118-*scrG*del, Km^r^	This work
pDZ_414	pRE118-VP0117del, Km^r^	This work
pDZ_154	pRE118-*cpsR*del, Km^r^	This work
pDZ_146	pRE118-*cpsS*del, Km^r^	This work
pRK2073	Derivative of the mobilization helper plasmid pRK2013. ColE1, RK2-Mob^+^, RK2-Tra^+^, Km^s^ Sp^r^ derivative of pRK2013	Leong et al. (59), Lourdes Girard
pRK600	Derivative of the mobilization helper plasmid pRK2013. ColE1, RK2-Mob^+^, RK2-Tra^+^, Km^s^ Cm^r^ derivative of pRK2013	60
pFY_1554 (pMMB67EH-Gm)	Expression vector harboring *lacI* and containing a Ptac promoter, Gm^r^	Samuel Miller, Fitnat Yildiz
pDZ_133	*cdgF* cloned in pFY_1554	This work
pFY_4535	C-di-GMP biosensor containing the *hok*/*sok* region from pXB300, Gmr	32
pDZ_119	C-di-GMP biosensor cloned in a pBBRMCS backbone, Cmr	27
pFY_691 (pBBRlux)	*luxCDABE*-based promoter fusion vector; Cm^r^	61
pDZ_92	pBBRlux-P*_tpdA_*; Cm^r^	27
pDZ_46	pBBRlux-P*_cpsA_*; Cm^r^	27

### Generation of genetic constructs.

The primers used for the generation of genetic constructs are described in Table 2. Genomic DNA from V. parahaemolyticus RIMD2210633 or V. cholerae A1552 was used as the template for the amplification of the regions of interest through PCR. PCR amplicons used for the generation of genetic constructs were produced by the high-fidelity DNA polymerase Q5 from New England BioLabs (Ipswich, MA). PCR purification and plasmid isolation was done using the DNA Clean & Concentrator-5 and Zyppy Plasmid Miniprep Kits from Zymo Research (Irvine, CA), respectively.

**TABLE 2:** List of primers used in this study

Primer no.	Sequence 5′–3′	Construct
DZ_125	caagcttcttctagaggtacATAAAATCAACTTGATGAGTCAG	pDZ_179
DZ_126	cgcgattgtaGTCCATATCCATTTTCCTTG	
DZ_127	ggatatggacTACAATCGCGAACACTAAAGC	
DZ_128	catgaattcccgggagagctAAGTGGCTTGGGTTGGTAAG	
DZ_364	caagcttcttctagaggtacatcgacgaagacgtcaag	pDZ_366
DZ_365	ggtttagaagaacgattattgcgcttatc	
DZ_366	aataatcgttcttctaaaccaacctacttg	
DZ_367	catgaattcccgggagagctatgtcttgagtaccacag	
DZ_318	caagcttcttctagaggtacTTTTTAATGCCTTCTGCG	pDZ_364
DZ_319	aatatcggctAATGAAGTAATCATGCGC	
DZ_320	ttacttcattAGCCGATATTCATTGAATTC	
DZ_321	catgaattcccgggagagctATTGTGAGTCTGCTGATG	
DZ_348	caagcttcttctagaggtaccagtcgaaattctcaatcg	pDZ_414
DZ_349	aatgtgttggacgtagtgtaatttgagtc	
DZ_350	tacactacgtccaacacatttggttaac	
DZ_351	catgaattcccgggagagctagtttgtgtttcattgcc	
DZ_75	caggaaacagaattcgagctGTGATGACAACTGAAGATTTCAAAAAATC	pDZ_133
DZ_76	cctgcaggtcgactctagagTTAGAGCGGCATGACTCG	
DZ_86	cgacggatcccaagcttcttCTCAGAGCTTAACGATGC	pDZ_154
DZ_87	cttgatcggaCTTAAACTGCCCAGCCATTC	
DZ_88	gcagtttaagTCCGATCAAGCGATGTAATTC	
DZ_89	catgaattcccgggagagctAAATGATCGACTGGGAAAAATAG	
DZ_82	cgacggatcccaagcttcttTAAGTACTATGAACGACTTCG	pDZ_146
DZ_83	agtgatctgaATTCCTTGTAGTTTGATGC	
DZ_84	tacaaggaatTCAGATCACTCTATAGAGATG	
DZ_85	catgaattcccgggagagctATAGTACCTAGCACTTTATTG	

The deletion constructs were assembled using the plasmid pRE118, which is a suicide plasmid in V. parahaemolyticus. The recombination substrates, used to eliminate endogenous genes through double homologous recombination, consisted of approximately 500 bp of upstream and downstream sequences flanking the site to be deleted. The plasmids were designed to generate in-frame deletions that eliminate more than 70% of the protein sequence of the product. Plasmid pRE118 was digested with the restriction enzymes SacI-HF and KpnI-HF. The upstream and downstream recombination substrates were fused with each other and to the linearized plasmid pRE118 through a modified isothermal assembly protocol, using the NEBuilder HiFi Assembly Master Mix. We prepared 6-μL reaction mixtures consisting of at least 50 ng of each DNA assembly part and 2 μL of NEBuilder HiFi Assembly Master Mix. Primers were designed with the assistance of the online NEBuilder Assembly Tool. The assembled constructs were selected after screening clones through colony PCR with the MyTaq Red polymerase mix from Meridian Bioscience.

For the overexpression of the DGC CdgF (VCA0956) from V. cholerae, we cloned a PCR product corresponding to the coding sequence of *cdgF* in the plasmid pMMB67EH-Gm. The plasmid was digested with the restriction enzymes SacI-HF and BamHI-HF. The PCR product was assembled into the linearized plasmid using the same isothermal assembly protocol described in the previous paragraph. Correct assemblies were identified through colony PCR as described above.

The genetic constructs were mobilized into the strains of interest through conjugation. For biparental mating, we used the donor strain SM10λ*pir*, which carries the conjugation machinery of the RP4 conjugative plasmid. For triparental mattings, we used a helper strain hosting the plasmid pRK2073 or the plasmid pRK600, which are derivatives of pRK2013 (48) and have the RK2 delivery machinery. To promote conjugation, we mixed the strains at a 1:1 ratio in LB broth, spotted 50 μL of the suspension over LB agar plates without antibiotics, and incubated them overnight at 37°C. The mating spots were recovered with a sterile pipette tip, resuspended in 1 mL LB broth, and serially diluted. The dilutions were plated on LB agar plates with antibiotics that select for the presence of the desired plasmid in the recipient strain.

### Generation of mutant strains with deletions of genes of interest.

Deletion of the genes of interest was performed as previously described (27), with minor modifications described below. Single colonies from single recombinant strains were grown in LB broth for approximately 8 h at 37°C with agitation (200 rpm). The cultures were then diluted (1:100) in LB broth and spread on LB agar plates supplemented with 10% sucrose. The rest of the genetic protocol was performed as previously described (27).

### Luminescence assay.

The luminescence assays in planktonic cultures were performed as previously described (27). To quantify light production in cells growing on LB agar, we poured 2 mL of LB agar per well with 5 μg/mL Chl into 12-well microplates and let them dry for 1 h with the lid open under sterile conditions, and for another 24 h with the lid closed at room temperature. Overnight cultures of the strains of interest were diluted (1:200) in fresh LB and 2 μL of each sample was placed in the middle of each well. Plates were incubated at 30°C for 24, 48, 72, and 96 h. The luminescence of each well was measured using the Synergy H1 plate reader (BioTek, Winooski, VT). To normalize light production by cell growth, we resuspended the spot-colonies of each well in 1 mL of fresh LB. The suspension was diluted (1:5) and the optical density at 600 nm (OD_600_) was measured with an Epoch 2 microplate reader (BioTek). The relative luminescent units (RLU) are calculated as arbitrary luminescence divided by the OD_600_ of the resuspended spot-colonies. Two independent experiments were performed with three biological replicates each.

To analyze luminescence from biofilms formed on glass tubes under shaking conditions, we first prepared overnight cultures of the strains of interest. The test tubes containing bacterial cultures were always set angled in a tube-rack inside a shaking incubator. The overnight cultures were diluted (1:1,000) in 5 mL LB broth with 5 μg/mL Chl and grown with agitation at 30°C for 24 h. Under these conditions, biofilms are formed a few centimeters above the planktonic culture. A sample of the planktonic culture was taken and diluted (1:5) to measure the OD_600_ as described above. The rest of the planktonic culture was carefully discarded, avoiding disruption of the biofilms. The test tubes with the biofilms were washed with 1 mL LB to remove the remaining planktonic culture. Biofilms were resuspended in 800 μL LB. Next, 200-μL samples of each resuspended biofilm were transferred to white, clear-bottomed 96-well plates. We measured the OD_600_ of the resuspended biofilms and their light emission using the Synergy H1 plate reader (BioTek). The RLU are calculated as arbitrary luminescence units per mL divided by the OD_600_ of the biofilm samples. Experiments were performed twice independently with three biological replicates each time.

### Determination of the relative abundance of c-di-GMP using a genetic c-di-GMP reporter.

The estimation of c-di-GMP levels in planktonic cultures using the c-di-GMP genetic reporter present in plasmids pFY4535 and pDZ119 was performed as described previously (27). We measured the autofluorescence of cells lacking the c-di-GMP reporter and used the average of three independent samples as a blank. To measure c-di-GMP levels using the c-di-GMP genetic reporter in cells grown on LB agar, we performed the same type of experiments as described above for the luminescence assays, with minor modifications. Overnight cultures of the strains of interest were diluted (1:200) in fresh LB and 2 μL of each sample was pipetted into the center of wells containing LB agar and 15 μg/mL Gen. Plates were incubated at 30°C for 24, 48, 72, 96, and 120 h. The fluorescence of the AmCyan and TurboRFP proteins produced by the spot colonies from each well was measured using a Synergy H1 plate reader (BioTek). We used the constitutive fluorescence of AmCyan to normalize TurboRFP production by reporter expression; hence, we were able to track fluorescence from the same spot colonies over time. We also measured the autofluorescence of cells lacking the c-di-GMP reporter growing on LB agar under the same conditions as described previously, and used the average of three independent samples as a blank. The relative fluorescence intensity was calculated by dividing the arbitrary fluorescence intensity units of TurboRFP by those of AmCyan. Three independent experiments were performed with a total of at least 5 biological replicates.

To analyze c-di-GMP levels from biofilms formed on glass tubes under shaking conditions using the c-di-GMP genetic reporter, we followed a similar experimental procedure to that previously described for the luminescence assays. We first grew overnight cultures of the strains of interest, diluted them (1:1,000) in 5 mL LB broth with 15 μg/mL Gen, and grew them again with agitation at 30°C for 24 h. The planktonic culture was carefully discarded, avoiding disruption of the biofilms. We washed the biofilms with 1 mL sterile distilled water to remove the remaining planktonic culture. We then resuspended the biofilms in 800 μL sterile distilled water and transferred a 200-μL sample to black 96-well plates. We measured the fluorescence of AmCyan and TurboRFP from resuspended biofilms using the Synergy H1 plate reader (BioTek). As described previously, we used the autofluorescence of biofilm cells lacking a fluorescent reporter as a blank.

### Liquid-solid interface biofilm assays over glass or a PVC surface.

Biofilms formed on glass under shaking conditions were grown as described above. Overnight cultures (5 mL) of the strains of interest were prepared in test tubes and placed angled in a tube-rack inside a shaking incubator. Afterwards, the cultures were diluted (1:1,000) in the same volume of LB and grown with agitation at 30°C for 24 h. The OD_600_ of the planktonic culture was measured using an Epoch or Synergy plate reader. The planktonic culture was carefully discarded, avoiding disturbance of the biofilm integrity. The tubes were washed once with 1 mL LB by pipetting to remove unattached cells at the bottom of the tube. The biofilm was resuspended in 800 μL LB and a 200-μL sample was used to measure the OD_600_. Biofilm formation values were obtained after dividing the OD_600_ of the biofilm cells by the OD_600_ of their corresponding planktonic culture.

Biofilm formation on PVC wells was performed using a previously reported crystal violet staining procedure with some modifications (49). The strains of interest were grown overnight in 5 mL LB. Cultures were then diluted to an OD_600_ of 0.02. Next, 150-μL samples of the cultures and a blank sample consisting of sterile LB broth were transferred to 96-well PVC microplates previously sterilized with UV light for 10 min. Wells were incubated for 2, 4, 6, and 8 h at 30°C under static conditions. To measure the growth of the planktonic cultures, we analyzed their OD_600_ using an Epoch2 microplate reader. The planktonic cultures were carefully discarded in a 10% bleach solution and washed twice with tap water by decantation. The wells were dried upside-down over paper towels, and the biofilms were then stained with a 0.1% crystal violet solution for 10 min at room temperature. To solubilize the dye retained by the biofilms, we added 150 μL of ethanol (absolute) to the wells and incubated them for approximately 30 min at room temperature under static conditions. We measured the OD_600_ of the solubilized crystal violet dye with an Epoch2 microplate reader. Biofilm formation values were obtained by dividing the OD_600_ of the dye retained by the biofilms by the OD_600_ of the planktonic cultures from the same well. At least three independent experiments were performed for each strain of interest.

### Bioinformatic analysis of the GSE122479 experiment from the GEO data sets.

We used the raw data of the GEO data sets under accession no. GSE122479, generated in a previous report (37). The analysis was performed by the Unidad de Análisis Bioinformáticos bioinformatic facility at the Center for Genomic Sciences (UNAM). The data were analyzed with FastQC v0.11.8 before trimming (https://www.bioinformatics.babraham.ac.uk/projects/fastqc/). The program trim_galore 0.6.6 (https://www.bioinformatics.babraham.ac.uk/projects/trim_galore/) was used to remove adaptor and other undesirable sequences. The obtained sequences were mapped to the reference genome (GCF_000196095.1_ASM19609v1) using the program Bowtie2 (50). The programs MACS2 and HOMER were used for peak calling.

The genes associated with the ChIP-seq peaks identified were mapped to genes in the KEGG pathway database (https://www.genome.jp/kegg/pathway.html) using KEGG Mapper (51, 52).

The sequences of peaks (or reverse complementary sequences, for genes codified in the negative strand) associated with genes mapped to the biofilm formation KEGG pathway or gene products predicted to be involved in c-di-GMP metabolism were used as seed to identify conserved motifs using the Multiple Em for Motif Elicitation (MEME) program (53, 54).
